# Supplementary material for: Identification of photoperiod-induced specific miRNAs in the adrenal glands of Sunite sheep (Ovis aries)
Source: Front Vet Sci. 2022 Jul 22;9:888207. doi: 10.3389/fvets.2022.888207 (PMC9354845; doi:10.3389/fvets.2022.888207)
Supplement: Supplementary file 1 [file Data_Sheet_1.ZIP › Supplementary file/miRDeep2 file.zip!/miRDeep2 file/Know_Structure/SPLP42A3/oar-mir-191.pdf]

miRBase precursor : oar-mir-191
Total read count : 47441
oar-miR-191 read count : 47230
remaining reads : 211

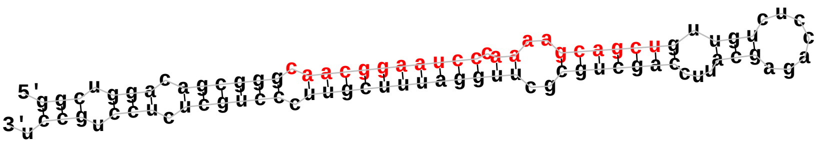

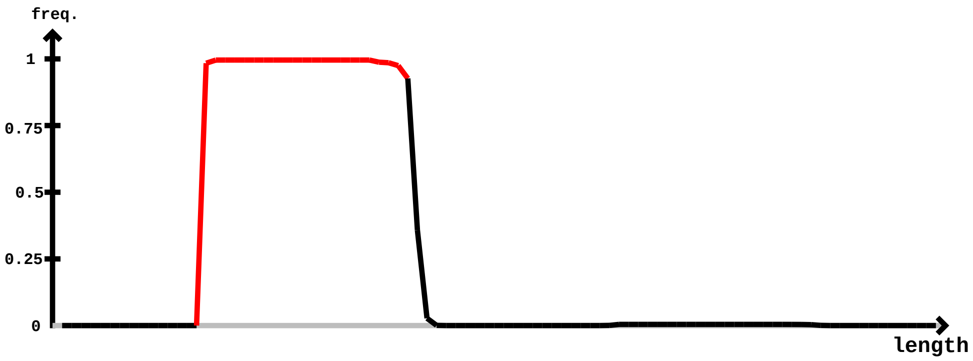

oar-miR-191

```
5'- ggcuggacagcgggcaacggaaucccaaaagcagcuguugucuccagagcauuccagcugcgcuuggauuucguucccugcucuccugccu   -3'   exp
    (((.(((.((((((.((((((((((.((..(((((((.(((.......)))...)))))))..))))))))))))).)))))).))).))).   reads   mm   sample
    ..............caacggaaucccaaGagc...........................................................   1       1    seq
    ..............caacggaaucccaaaagc...........................................................   376     0    seq
    ..............caacggaaucccaaaagca..........................................................   102     0    seq
    ..............caacggaaucccaaaagcag.........................................................   508     0    seq
    ..............caacggaaucccaaaagcagc........................................................   2276    0    seq
    ..............caacggaaucccaaaagcagcu.......................................................   26802   0    seq
    ..............caacggaaucccaaaagcagcug......................................................   15381   0    seq
    ..............caacggaaucccaaaagcagcugu.....................................................   1192    0    seq
    ..............caacggaaucccaaaagcagcuguu....................................................   33      0    seq
    ...............aacggaaucccaaaagcagc........................................................   9       0    seq
    ...............aacggaaucccaaaagcagcu.......................................................   168     0    seq
    ...............aacggaaucccaaaagcagcug......................................................   306     0    seq
    ...............aacggaaucccaaaagcagcugu.....................................................   73      0    seq
    ...............aacggaaucccaaaagcagcuguu....................................................   1       0    seq
    ................acggaaucccaaaagcagcug......................................................   2       0    seq
    .......................................................agcugcgcuuggauuucguuccc.............   1       0    seq
    ........................................................gcugcgcuuggauuucguu................   3       0    seq
    ........................................................gcugcgcuuggauuucguuc...............   2       0    seq
    ........................................................gcugcgcuuggauuucguucc..............   10      0    seq
    ........................................................gcugcgcuuggauuucguuccc.............   12      0    seq
    .........................................................cugcgcuuggauuucguu................   4       0    seq
    .........................................................cugcgcuuggauuucguuc...............   9       0    seq
    .........................................................cugcgcuuggauuucguucc..............   23      0    seq
    .........................................................cugcgcuuggauuucguuccc.............   114     0    seq
    .........................................................cugcgcuuggauuucguucccu............   32      0    seq
    ..........................................................ugcgcuuggauuucguucccu............   1       0    seq
```
